# Isolation by distance, web service

**DOI:** 10.1186/1471-2156-6-13

**Published:** 2005-03-11

**Authors:** Jeffrey L Jensen, Andrew J Bohonak, Scott T Kelley

**Affiliations:** 1 High Tech High, 2861 Womble Road, San Diego, CA 92106-6025, USA; 2 Department of Biology, 5500 Campanile Drive, San Diego State University, San Diego, CA 92182-4164, USA

## Abstract

**Background:**

The population genetic pattern known as "isolation by distance" results from spatially limited gene flow and is a commonly observed phenomenon in natural populations. However, few software programs exist for estimating the degree of isolation by distance among populations, and they tend not to be user-friendly.

**Results:**

We have created Isolation by Distance Web Service (IBDWS) a user-friendly web interface for determining patterns of isolation by distance. Using this site, population geneticists can perform a variety of powerful statistical tests including Mantel tests, Reduced Major Axis (RMA) regression analysis, as well as calculate *F*_*ST *_between all pairs of populations and perform basic summary statistics (e.g., heterozygosity). All statistical results, including publication-quality scatter plots in Postscript format, are returned rapidly to the user and can be easily downloaded.

**Conclusion:**

IBDWS population genetics analysis software is hosted at  and documentation is available at . The source code has been made available on Source Forge at .

## Background

The term "isolation by distance" (IBD) was first used by Sewall Wright [1,2] to describe patterns of population genetic variation that derive from spatially limited gene flow. IBD is defined as a decrease in the genetic similarity among populations as the geographic distance between them increases. Statistical tests for IBD can be conducted using populations or individuals as the units of replication, although analyses at the individual level typically utilize spatial autocorrelation statistics [3]. When individuals can be grouped into populations, it is possible to calculate 1) a matrix of genetic similarity or distance between all population pairs for comparison with 2) a matrix of pairwise geographic distances. A nonparametric Mantel test is typically used to test for non-random associations between these two matrices. Bohonak [4] has also suggested performing statistical tests of the IBD slope and intercept using bootstrapped pseudoreplicates, and recently, Yang [5] has developed a likelihood-based approach for IBD analysis. The ever-growing volume of population genetic data and a continued desire to interpret spatial processes make computational tools for rapidly identifying patterns of isolation by distance highly desirable.

In this paper we describe a sophisticated, yet user-friendly, Common Gateway Interface (CGI) implementation of statistical software for detecting IBD patterns. Isolation by Distance Web Service (IBDWS) is a major expansion and upgrade of Isolation By Distance (IBD), a standalone computer program written in C and originally compiled for Windows and Macintosh [4]. IBDWS includes the following highly useful features for analysis of genetic structure in natural populations:

(1) Analysis of raw data: For a raw data set with codominant markers (e.g., microsatellites), IBDWS calculates basic summary statistics including heterozygosity, *F*_*ST *_between all pairs of populations, and standard transformations of *F*_*ST*_.

(2) Statistical tests: IBDWS performs Mantel tests, partial Mantel tests, reduced major axis (RMA) regression of IBD slope and intercept, and error estimation for slope and intercept using multiple methods.

(3) Simple form interface: Various parameters (e.g., number of randomizations) are easily configured on the IBDWS front page and are checked for validity.

(4) Analysis selection: Multiple analyses may be performed via checkbox selections.

(5) Publication quality graphics: IBD scatter plots are generated in Postscript format for download and further modification.

(6) Rapid analysis: IBDWS is hosted on a fast server with minimal wait times (see Results and discussion).

The majority of these features were not included in IBD. Additionally, IBDWS includes thirty-one updates and bug fixes to the original IBD statistical package. Altogether, these changes make IBDWS a major advancement on the original release in terms of both performance and utility.

## Implementation

IBDWS was written in C++, compiled with the Unix g++ compiler, and runs on a server using Apache 2.0.50 and Fedora Linux. Postscript file plotting capability (see below) was implemented using CGraph, a C++ plotting library. The UNIX command "convert" is called by IBDWS to create a JPEG for web-based viewing from the postscript file. Both files are held on the server for 24 hours until routine deletion.

**Figure 1:**
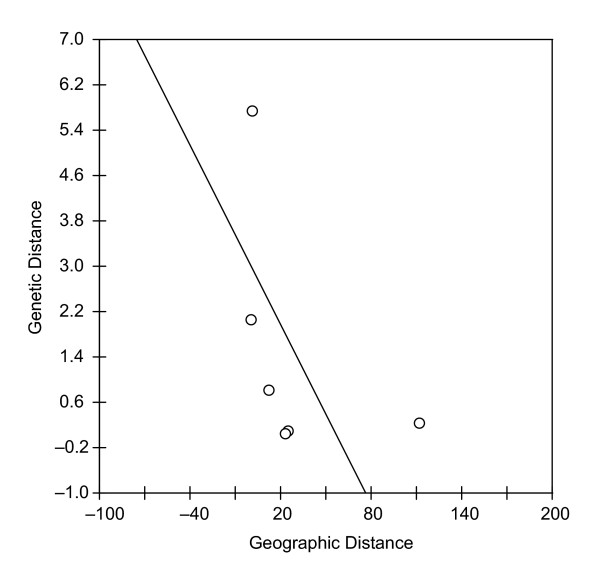
**Example scatter plot. **The plot was generated using the example raw allelic data and geographic information from the IBD.2 example data set available on the IBDWS website. The RMA regression line overlays the scatter plot. The plot was downloaded in postscript format from the website and slightly modified using Adobe Illustrator.

Depending on the specific configuration options, IBDWS performs four primary functions. The first is generating standard summary statistics from raw data (if provided). These include a matrix of the genetic distance *F*_*ST *_[1] between all pairs of populations, and several standard transformations of this statistic [see [6,7]]. Second, the scatter plot of genetic vs. geographic distance is generated in JPEG format (for viewing on the web page) and in vector-based Postscript format (for downloading and manipulating in a graphics design program). The Reduced major axis regression line overlays the plot (see Figure 1). An algorithm that evaluates the range of values on each axis chooses an appropriate set of evenly spaced units for labelling.

**Figure 2:**
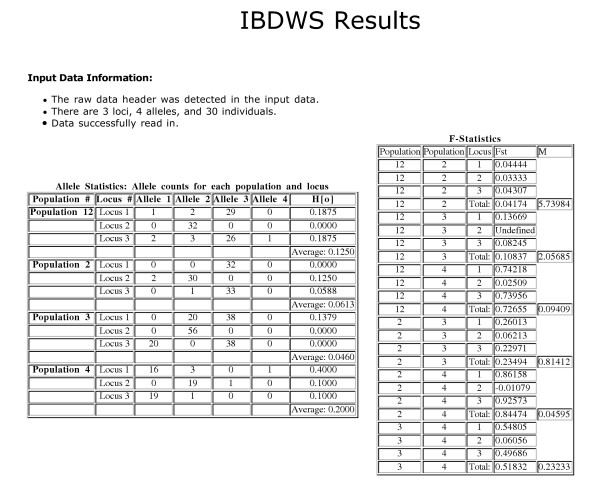
**IBDWS example statistical results output. **The figure shows a partial list of statistical output calculated from the IBD.2 sample data set. Results of Mantel tests and other calculations are also provided. All results can be downloaded as text files.

Third, IBDWS assesses whether there is a statistically significant relationship between the genetic distance (or similarity) matrix and the comparable matrix of geographic distances. As in other software applications, a Mantel test [8] is used to test this relationship, as it properly accounts for the population as the unit of replication, rather than population pairs (i.e., the matrix entries). In addition, IBDWS calculates the slope and intercept of the IBD relationship using reduced major axis (RMA) regression. RMA regression is more appropriate than standard linear regression when both the dependent and the independent variables are measured with error. Error for the slope and intercept are estimated using standard linear approximations, using a one-delete jackknife across population pairs, and by bootstrapping pseudoreplicates over *independent *population pairs (see [4] and the online IBD manual). The final two methods are preferred, as they correctly treat the population as the unit of replication. Figure 2 provides examples of IBDWS statistical output.

Different researchers have recommended logarithmic (base 10) transformation of genetic distance, geographic distance or both, prior to IBD analysis [6,9]. IBDWS automatically performs each of these transformations and provides an interface that switches among the four possible scatter plots.

## Results and discussion

### Input

**Figure 3:**
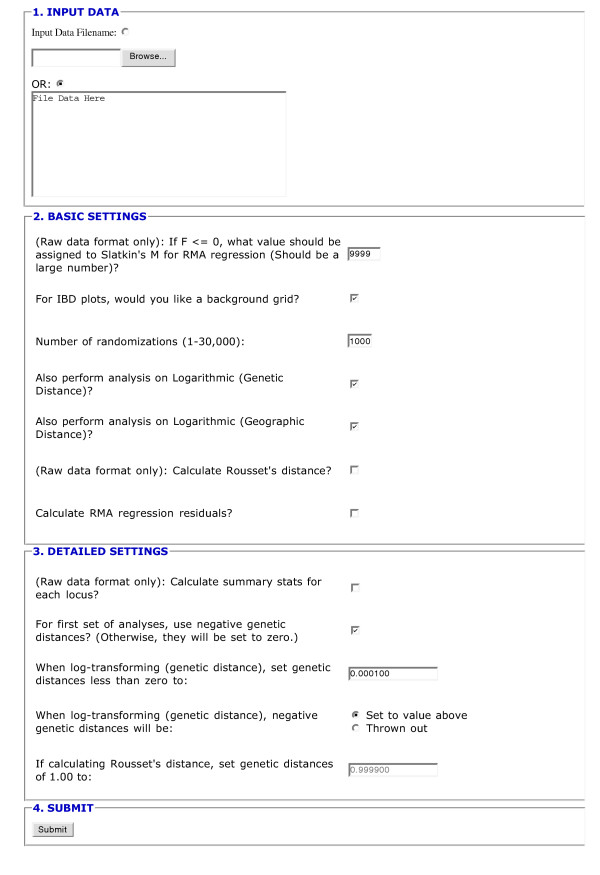
**IBDWS forms interface. **The figure shows a portion of the CGI web interface. Data can be uploaded from a text file using the Browse button or pasted directly into the form. Below the data input portion is a partial list of checkboxes for tailoring analyses. A total of twelve parameters are modifiable by the user.

IBDWS analyzes genetic data with or without corresponding geographical information. Without geographic distances, summary statistics can be calculated from raw data sets that include diploid genotypes at one or more loci. These statistics include heterozygosity, and estimates of genetic similarity between all population pairs. The additional input of geographic distance between all population pairs facilitates the variety of IBD calculations described above. The geographic distance matrix can be calculated using simple Euclidean distances, or with more complex algorithms that follow biologically relevant landscape features. Input data can be uploaded as an ASCII text file or the data can be inserted into an HTML form on the IBDWS web page (Figure 3). White space delimiters are used between fields. If the population genetic distance matrix is entered directly (rather than individual-level data), the input file may be in matrix or list format (examples of each are provided on the IBDWS home page). Indicator labels (e.g., GENETIC_DISTANCE, GEOGRAPHIC_DISTANCE) are required to separate blocks in the input file or test string, assuring that it is parsed correctly. Additional documentation regarding input for partial Mantel tests is provided in the IBD manual (available at the web site).

Checkboxes and radio buttons allow the user to configure which analyses will be performed (Figure 3). These include the calculation of Rousset's genetic distance [7] from raw data, options to log-transform genetic and/or geographic distances, replacement values for undefined transformations, and the number of bootstrap randomizations per analysis (up to 30,000).

### Output

A full analysis of raw data for six loci, two to three alleles per locus, and 326 individuals required an average wait time of 10 seconds. When an input without any geographic distances is used, wait times are negligible. If raw genotypic data are supplied, IBDWS returns an HTML table with basic summary statistics, the genetic distance *F*_*ST *_between all population pairs, the genetic similarity statistic M [6] between all population pairs, and Rousset's [7] distance *F*_*ST *_/ (1 - *F*_*ST*_) if desired. If geographic distances are included, IBDWS will display a scatter plot of genetic distance vs. geographic distance for all pairs of populations, and overlay the RMA regression line (Figure 1). If log-transformation of the data is selected, a pull-down menu below the plot allows the user to choose data sets with one or both axes log-transformed. Clicking on any plot downloads a Postscript version to the user's computer. Statistical analyses of the IBD relationship, including Mantel tests, partial Mantel tests (if appropriate) and the RMA regression analyses described above, are summarized in HTML tables as well. A summary of all analyses can downloaded as a static HTML file, or a tab-delimited text file suitable for importation into a spreadsheet application.

### Future developments

At present, IBDWS requires the user to calculate all pairwise genetic distances between populations for DNA data sets. In future versions, we plan to include algorithms for calculating genetic distances using both sequence and allelic data, including genetic distances based on phylogenetic trees under different models of evolution. When implemented, the user will simply have to supply the raw data and geographic distances before calculations are performed. IBDWS will also be updated with new population genetics analyses as they become available.

## Conclusion

In summary, IBDWS is a substantial expansion of the previous PC-based versions of IBD, providing geneticists with a simple interface, multiple statistical calculations, rapid analysis, and high-quality graphics. It will be useful to a significant number of molecular ecology, conservation genetics, population genetics and genomics researchers, and will be updated with newer analyses in the future.

## Availability and requirements

IBDWS is currently hosted at . Documentation of the statistical calculations and biological context are available on IBD's original website at . The programming source files have been made available at  under the GPL (GNU Public License).

## List of abbreviations used

IBD: Isolation by distance.

IBDWS: Isolation by distance web service.

RMA: Reduce major axis (regression).

## Authors' contributions

JLJ designed and implemented IBDWS. This effort included creating the CGI, debugging and enhancing IBD, implementing postscript graphing libraries and extensive testing of the software. JLJ also wrote the initial draft of the manuscript. AJB wrote the original IBD software, the precursor to IBDWS. AJB also upgraded the code, added graphing algorithms, provided technical advice and assistance to JLJ and contributed to the writing of the manuscript. STK was involved in the early training of JLJ for the project as a mentor in the High Tech High Internship program. STK also guided the development of the project, provided technical advice, and contributed substantially to the writing of the manuscript. All authors read and approved the final manuscript.
